# Using adenosine triphosphate bioluminescence level monitoring to identify bacterial reservoirs during two consecutive *Enterococcus faecium* and S*taphylococcus capitis* nosocomial infection outbreaks at a neonatal intensive care unit

**DOI:** 10.1186/s13756-023-01273-5

**Published:** 2023-07-13

**Authors:** Ye Ji Kim, Min Yeong Hong, Hyun Mi Kang, Sook Kyung Yum, Young Ah Youn, Dong-Gun Lee, Jin Han Kang

**Affiliations:** 1 Department of Pediatrics, College of Medicine, Seoul St. Mary’s Hospital, The Catholic University of Korea, Seoul, Republic of Korea; 2 Infection Control Office, Seoul St. Mary’s Hospital, Seoul, Republic of Korea; 3 Vaccine Bio Research Institute, College of Medicine, The Catholic University of Korea, Seoul, Republic of Korea; 4 Division of Infectious Diseases, Department of Internal Medicine, College of Medicine, The Catholic University of Korea, Seoul, Republic of Korea

**Keywords:** Neonatal intensive care units, Outbreak, *Enterococcus faecium*, *Staphylococcus capitis*, Infection source identification, Infection control

## Abstract

**Introduction:**

This study aimed to assess the role of adenosine triphosphate (ATP) bioluminescence level monitoring for identifying reservoirs of the outbreak pathogen during two consecutive outbreaks caused by *Enterococcus faecium* and *Staphylococcus capitis* at a neonatal intensive care unit (NICU). The secondary aim was to evaluate the long-term sustainability of the infection control measures employed one year after the final intervention measures.

**Methods:**

Two outbreaks occurred during a 53-day period in two disconnected subunits, A and B, that share the same attending physicians. ATP bioluminescence level monitoring, environmental cultures, and hand cultures from healthcare workers (HCW) in the NICU were performed. Pulsed-field gel electrophoresis (PFGE) typing was carried out to investigate the phylogenetic relatedness of the isolated strains.

**Results:**

Four cases of *E. faecium* sepsis (patients A-8, A-7, A-9, B-8) and three cases of *S. capitis* sepsis (patients A-16, A-2, B-8) were diagnosed in six preterm infants over a span of 53 days. ATP levels remained high on keyboard 1 of the main station (2076 relative light unit [RLU]/100 cm^2^) and the keyboard of bed A-9 (4886 RLU/100 cm^2^). By guidance with the ATP results, environmental cultures showed that *E. faecium* isolated from the patients and from the main station’s keyboard 1 were genotypically indistinguishable. Two different *S. capitis* strains caused sepsis in three patients. A total 77.8% (n = 7/9) of *S. capitis* cultured from HCW's hands were genotypically indistinguishable to the strains isolated from A-2 and A-16. The remaining 22.2% (n = 2/9) were genotypically indistinguishable to patient B-8. Three interventions to decrease the risk of bacterial transmission were applied, with the final intervention including a switch of all keyboards and mice in NICU-A and B to disinfectable ones. Post-intervention prospective monitoring up to one year showed a decrease in blood culture positivity (*P* = 0.0019) and catheter-related blood stream infection rate (*P* = 0.016) before and after intervention.

**Conclusion:**

ATP monitoring is an effective tool in identifying difficult to disinfect areas in NICUs. Non-medical devices may serve as reservoirs of pathogens causing nosocomial outbreaks, and HCWs' hands contribute to bacterial transmission in NICUs.

**Supplementary Information:**

The online version contains supplementary material available at 10.1186/s13756-023-01273-5.

## Introduction

Neonates in neonatal intensive care units (NICUs) are vulnerable to nosocomial infections, which in turn are associated with increased infection-related morbidities and mortalities. The incidences and outcomes of these infections are determined by the type of pathogen and its antimicrobial susceptibility patterns, accompanying underlying medical conditions or degree of immaturity of the neonate’s immune system, and the number and type of invasive procedures that the neonate is exposed to. Furthermore, frequent and continuous contact with healthcare workers (HCWs) can be a source or mode of transmission of hospital acquired infections (HAIs) (1). Therefore, infection control policies practiced by the unit are extremely important in reducing rates of HAIs.

In order to terminate an outbreak, extensive investigations are generally carried out to identify the source of outbreak by performing environmental cultures and screening the patients as well as HCWs. However, in a review of 276 NICU outbreaks, only 48.6% of the attempts have succeeded in identifying the source of outbreak (2). Because HAIs in the NICU are associated with poor outcome of the neonates as well as increased length of admission duration leading to additional healthcare costs (3), preventing these infections are a priority.

Adenosine triphosphate (ATP) bioluminescence testing is useful to assess environmental cleanliness in environments with very low microbial counts and provides rapid feedback allowing immediate changes in cleaning practices (4). No consensus exists on objectively measuring environmental cleanliness within hospitals, especially depending on the location, risk of patients acquiring hospital acquired infections, and procedures or activities going on in the area. Therefore, alternative methods have been developed for assessing environmental cleanliness, including the ATP bioluminescence assay (5, 6). The level of ATP measures the quantity of organic matter, however, because it cannot discriminate between viable bacteria nor distinguish potential pathogens, many studies have expressed doubts about the usefulness of bioluminescence for hygiene monitoring within healthcare settings (7). However, a review on ATP-bioluminescence in healthcare environments proposed a cutoff of 100 relative light unit (RLU) /100 cm^2^ in high risk environments (8).

To effectively prevent and control infectious disease outbreak in the NICU, a systematic approach and assessment of all possible sources, reservoirs, and modes of transmission of the pathogen must be examined. Therefore, the primary aim of this study was to assess the role of ATP bioluminescence level monitoring for effectively identifying possible sources or reservoirs of the outbreak pathogen and find modes of transmission during the two consecutive outbreaks caused by *Enterococcus faecium* and *Staphylococcus capitis.* The secondary aim was to evaluate the long-term sustainability of the infection control measures employed one year after the outbreak termination by investigating changes in the rate of hospital-acquired sepsis and hospital-acquired catheter-related blood stream infections (CRBSIs).

## Methods

### Study setting

**Fig. 1:**
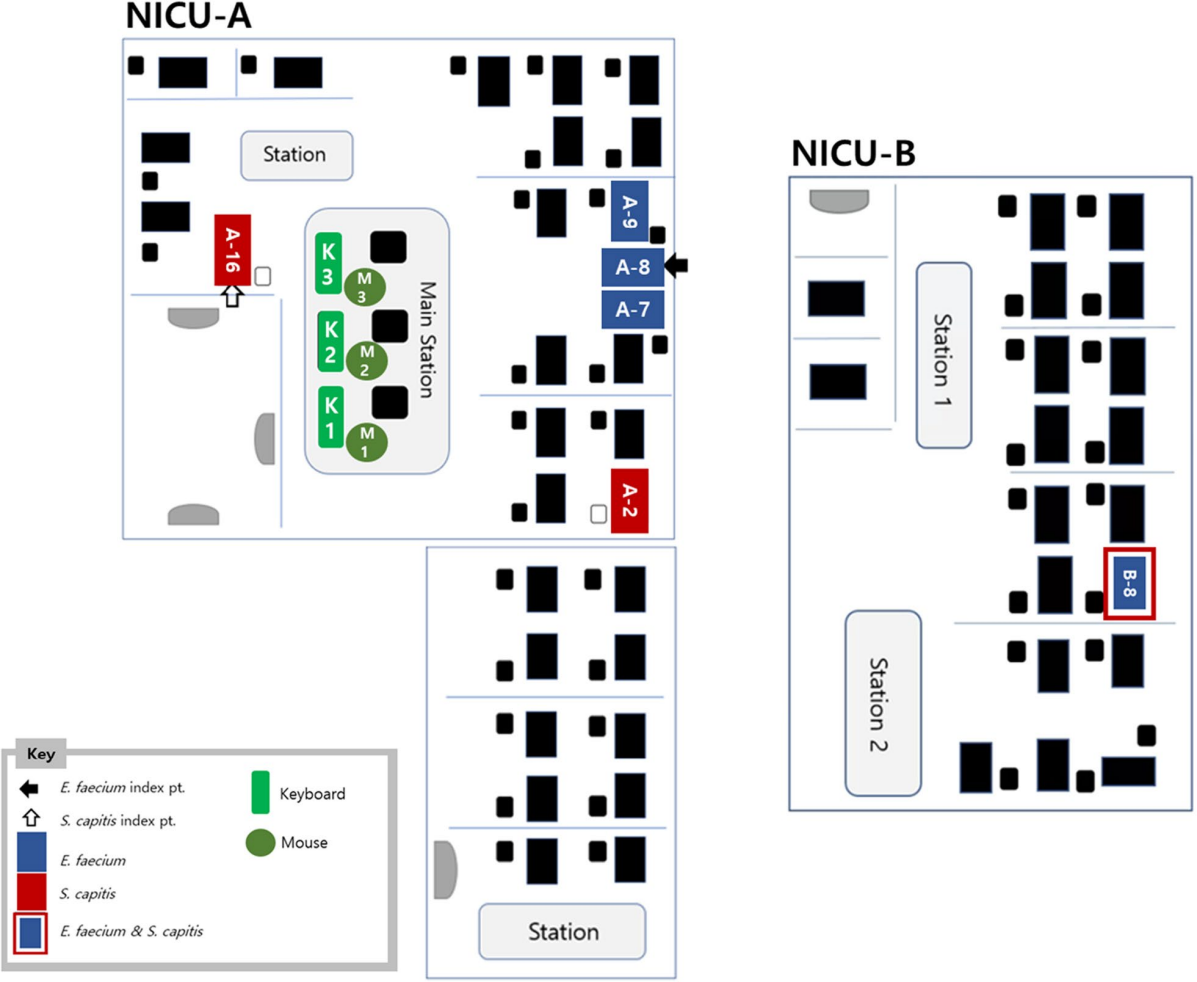
NICU patient placement map of Seoul St. Mary’s hospital. *E. faecium* outbreak occurred in neonates directly adjacent to the index patient, and then an additional sepsis case occurred in a patient located in the separated unit B. *S. capitis* outbreak occurred to neonates in unit A who were far apart from each other, and then the outbreak spread to unit B. M, mouse; NICU, neonatal intensive care unit; K, keyboard

Two consecutive outbreaks by different nosocomial bacteria—*E. faecium* and *S. capitis*—occurred during a span of 53 days at an NICU consisting of a total of 50 beds, of which 30 beds comprise subunit A and 20 beds comprise subunit B. The two subunits are located on opposite ends of the same floor with a hallway in between the two enclosed subunits which do not share on-duty nursing staff. However, they share the same attending physicians including neonatologists, residents, and interns. A nursery room for healthy newborns consisting of 20 bassinets are located in a separate area without any connections to the NICU. Sick or at-risk neonates are admitted directly after birth into either the NICU subunit A or B. Neonates are often transported between the nursery room and NICUs according to their medical needs, such as close monitoring or further evaluation and management (Fig. 1). A total of 75 resident nurses, 13 nursing assistants, 2 janitors, 4 residents, and 2 interns are assigned to NICU subunits A and B.

The routine cleaning protocol of the NICU before the outbreaks was done by cleaning staffs twice a day, during the day and evening duty. The disinfection of the surfaces around the patients’ incubator and medical devices was done once a day using Biospot® (Hydrachem, West Sussex, England), where one Biospot® effervescent chloride tablet was dissolved in 1.5 L of clean water making an overall concentration of 200 ppm. All the surfaces of medical devices and floors around each incubator were soaked for 3 min and swiped off using disposable towels or naturally air dried.

### Case definition

Sepsis was diagnosed in a patient with symptoms and/or signs of systemic infection plus *E. faecium* or *S. capitis* cultured from at least two peripheral blood cultures taken from different sites. Hospital-acquired infections were defined as positive cultures after 48 h of admission. Blood culture positivity rate was a percentage of the number of positive blood cultures divided by the total number of blood cultures tested in patients with symptoms and/or signs of systemic infection (with an onset of least 48 h after admission). Catheter-related blood stream infection (CRBSI) was diagnosed based on (1) a positive catheter tip culture or a positive blood culture drawn from the central venous catheter, (2) clinical symptoms and/or signs of systemic infection, and (3) a positive peripheral blood culture (9). The CRBSI rate was calculated by episodes per 1000 catheter days, and the *p* for trend was calculated. All patients with *E. faecium* and *S. capitis* sepsis had indwelling peripherally inserted central venous catheters that were unable to have blood drawn from.

### Interventions to decrease the risk of bacterial transmission

The first intervention to decrease the risk of bacterial transmission consisted of increasing the frequency of environmental disinfection from once daily (every 24 h) to three times a day (every 8 h).

The second intervention was to change the method and type of disinfection from Biospot® (as described in the study setting) to ready-made wipes (single-step detergent and disinfectant) to reduce any possibilities of mixing impurities in the dissolution process: Clinell® (GAMA Healthcare Ltd., Hemel Hempstead, England) (1000 ppm) was used on non-invasive medical devices such as ultrasonic devices, stethoscopes, etc., whereas, Safecide® (Cosell, Hanam, South Korea) (200 ppm) was used on all other surfaces.

The third and final intervention measures were: (1) changing keyboards and mice to disinfectable ones and (2) education of the HCWs on hand hygiene.

### Environmental surveillance

Environmental surface Adenosine triphosphate (ATP) bioluminescence levels were monitored using LUMITESTER PD-30 and LuciPac® Pen (R-Biopharm AG, Darmstadt, Germany). ATP monitoring consisted of checking ATP levels on environmental surfaces and medical devices at random visits by the infection control office during the day and evening shifts. The time of ATP measurement was randomly done during the day, prior to the disinfection. The tested area was 100cm^2^ including high-touched area around the patients’ incubator, medical devices, and also the surface of non-medical devices. Although the manufacturer defines a negative (clean) sample as a RLU level < 200 RLU/100 cm^2^ (10), in this study, all surfaces with ≥ 100 RLU/100 cm^2^ after disinfection were considered contaminated and thus re-cleaned, based on a review on ATP-bioluminescence in healthcare environments which proposed a cutoff of 100 RLU/100cm^2^ in high risk environments (8).

Environmental surface or medical device surface cultures were carried out on (1) areas with high ATP levels even after disinfection by protocol, (2) standardized environmental cultures from the patients’ surroundings (incubator handles, crash cart handles, stethoscopes, ventilator dials, infusion pump buttons, computer monitor buttons, keyboards, and mouse, (3) high-touch areas by HCW’s including keyboard and mice at the main station, with a total n = 49 environmental samples taken.

After the ATP measurements were made, the cleaning staffs and also the HCWs were given feedback on cleaning efficacy based on ATP level monitoring in order to improve environmental cleaning to decrease in the risk of environmental contamination consequently reducing the risk of pathogen transmission to bring forth a reduction in sepsis events.

### Hand washing surveillance

Hand washing and hand sanitization monitoring was carried out via unannounced visits by validated and trained observers. New NICU staff members were given hand hygiene education, all workers were re-educated on hand hygiene, and feedbacks on hand hygiene monitoring were given after each monitoring. Rectal swab cultures from the patients with *E. faecium* sepsis were also performed.

### Bacterial isolation, identification, and antibiotic susceptibility testing

Rectal swab cultures from the patients with *E. faecium* sepsis were also performed. All *E. faecium* and *S. capitis* isolated from patients with sepsis, healthcare workers’ hand cultures, and environmental cultures underwent species identification. Antibiotics susceptibilities were found using the automated microbiology system MicroScan Pos Breakpoint Combo Panel 44 (Siemens Healthcare Diagnostics Inc., West Sacramento, CA, USA). Pulsed-Field Gel Electrophoresis (PFGE) was carried out on the isolates (11), and *SmaI* PFGE patterns dendrogram was used to investigate the phylogenetic relatedness of the strains (12, 13).

### Methods of environmental and hand cultures

Hand cultures of physicians, nurses, and nurses’ aides that were on duty were performed on day 36 and 42. A total 29 HCWs’ hand cultures were included. Methods for hand cultures were done as follows: tryptic soy broth was poured on the hands of the workers and collected into 50 mL falcon tubes. The tubes were then incubated in 35 ± 2 °C for 18–24 h. The bacterial colonies grown were subculture in blood agar plates. For environmental cultures, environmental surfaces were rubbed with and aseptic swab and streaked onto blood agar plates. The plates were also incubated in 35 ± 2 °C for 18–24 h.

Species identification and antibiotic susceptibility tests were done via MALDI-TOF VITEK® MS (bioMerieux, Marcy L’Etoile, France) and VITEK®-2 (bioMerieux, Marcy L’Etoile, France).

### Statistics

The chi square test was used to calculate the significance of the change in sepsis rate before and after interventions. Interrupted time series analysis model was used to observe significance in variance between time periods before and after the intervention. All tests were two-tailed, and a *P* < 0.05 was considered statistically significant.

### Ethical statement

The Institutional Review Board (IRB) of Seoul St. Mary's Hospital approved this study (IRB No. KC22RISI0690). Informed consent was waived due to retrospective study design.

## Results

### Description of the outbreaks

**Fig. 2:**
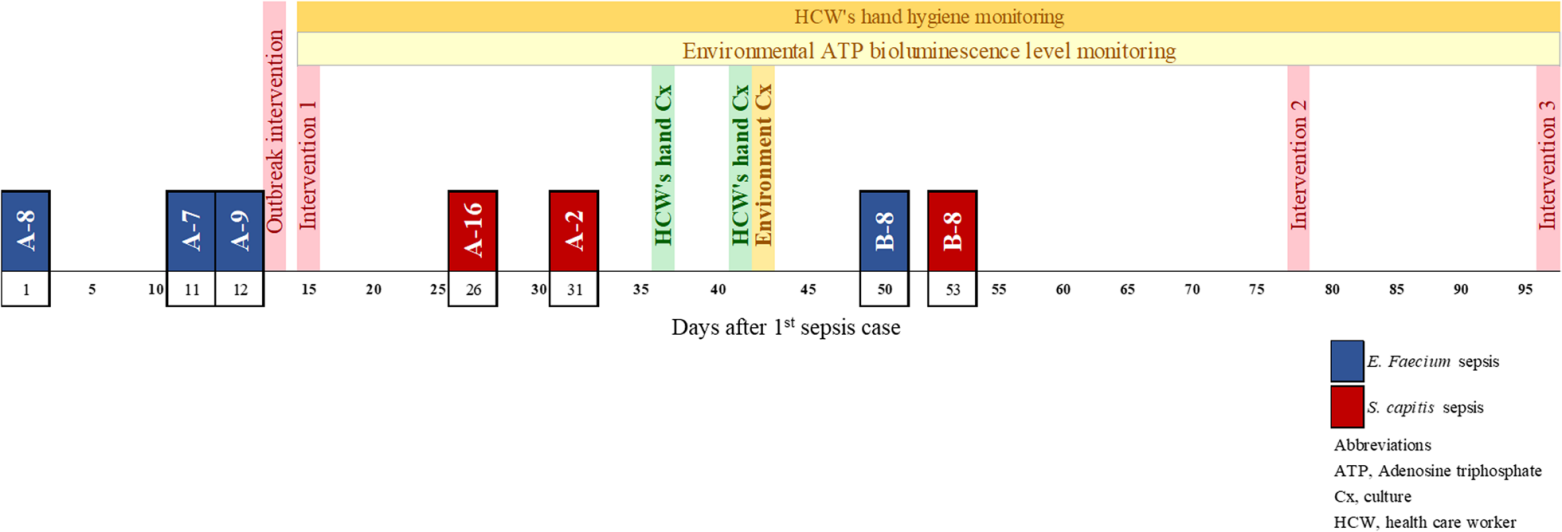
Timeline of the outbreak and intervention measures carried out. The outbreak was recognized on day 13, and intervention measures were applied after recognition. The first intervention consisted of increasing the frequency of environmental disinfection from once daily (every 24 h) to three times a day (every 8 h), the second intervention was to change the type of disinfectant from Biospot® to Clinell® and Safecide®. The final intervention consisted of changing keyboards and mice to disinfectable ones and education of the HCWs on hand hygiene based on the PFGE typing results. *HCW* health care worker; *PFGE* pulsed-field gel electrophoresis.

An *E. faecium* outbreak was first suspected when three preterm infants were diagnosed with *E. faecium* sepsis over a span of 12 days. The index case of the *E. faecium* outbreak was a preterm infant (A-8) born at gestational age 25 weeks and 4 days with a birthweight of 761 g. This infant was intubated and was diagnosed with necrotizing enterocolitis grade IIIB and therefore underwent double barrel ileostomy small bowel segmental resection one day prior to *E. faecium* sepsis, which occurred at 21 days of birth (Fig. 2). On day 26 after the *E. faecium* sepsis index case occurred, the *S. capitis* outbreak’s index case (A-16) was diagnosed with *S. capitis* sepsis. This preterm infant born at gestational age 29 weeks and 1 day with a birthweight of 1319 g had been diagnosed with respiratory distress syndrome (RDS) and patent ductus arteriosus (PDA), two weeks prior. This patient was diagnosed with *S. capitis* sepsis 21 days after birth. In total, four cases of *E. faecium* sepsis (A-8, A-7, A-9, B-8) and three cases of *S. capitis* sepsis (A-16, A-2, B-8) were diagnosed in 6 preterm infants over a span of 53 days (Fig. 2). The antibiotic susceptibility patterns of *E. faecium* and *S. capitis* cultured from the patients are on Additional file 1: A.

There were no sepsis episodes or CRBSIs caused by *E. faecium* or *S. capitis* within a 3-month period prior to the index cases. After investigations of the outbreaks and three interventions to reduce the risks of bacterial transmission (Fig. 2), no further cases of sepsis caused by *E. faecium* occurred during the one-year monitoring period after the final intervention and no more cases of *S. capitis* CRBSI occurred up to three months after the final intervention.

### Description of infection control measures

The following outbreak response measures were carried out to identify the source of infection and prevent further transmission:

#### 1) Alerting the hospital’s infection control office

The hospital’s infection control office was notified of a possible *E. faecium* outbreak on day 13 of the outbreak, after the identification of the third case of *E. faecium* sepsis. Together with the infection control office and NICU quality control team, outbreak management and three intervention measures were applied.

#### 2) Investigation of possible sources and reservoirs in the environment

ATP levels were found to be especially high on keyboards of the computers designated to the beds of the patients with *E. faecium* sepsis, handles of the crash carts, and fluid infusion pump buttons (Table 1). Therefore, the first intervention of increasing the frequency of environmental disinfection was applied (Fig. 2). The disinfection areas included all the surfaces around patients’ surroundings, medical devices (e.g. ventilator) and also non-medical devices (e.g. desks, keyboards, mouse in the station, drug drawer, milk room desks, buttons of intercom etc.). After the increase in the frequency of environmental disinfection, follow-up random ATP levels on day 21 of the outbreak showed that the levels on the keyboards of bed A-8 (536 RLU/100 cm^2^) and A-9 (530 RLU/100 cm^2^), as well as the handle of crash cart by bed A-9 (785 RLU/100 cm^2^) remained high while the levels on other sites had decreased significantly (Table 1).

**Table 1:**
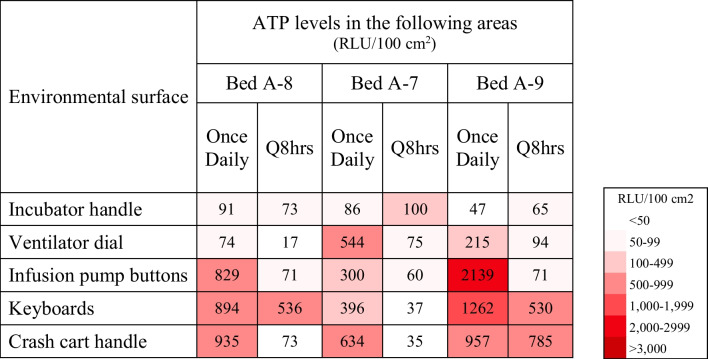
Random environmental surface ATP bioluminescence levels during once daily versus every 8-h environmental disinfection protocols during the *Enterococcus faecium* outbreak in NICU subunit A (Intervention 1)

All surfaces with ≥ 100 RLU/100 cm^2^ after disinfection were considered contaminated and thus re-cleaned

ATP adenosine triphosphate; NICU neonatal intensive care unit; RLU relative light units

Because increasing the frequency of environmental disinfection was not enough to maintain a clean environment, the second intervention of changing the method and type of disinfectant was incorporated. The surface of areas such as keyboards, mice, handles of the crash carts, and ventilator buttons needed to be soaked for 3 min to be effectively disinfected with the current method of disinfection, however, this was considered to be difficult. Therefore, intervention 2 consisted of using ready-made wipes, Clinell® and Safecide® to wipe the surfaces without the need for soaking.

Even after the second intervention, follow up ATP levels at random times during the day remained very high on keyboard 1 of the main station (2076 RLU/100 cm^2^) and the keyboard of bed A-9 (4886 RLU/100 cm^2^). In addition, a high level of ATP was detected on the handle of the crash cart by bed A-2 (1307 RLU/100 cm^2^) (Table 2). In parallel to ATP monitoring, cultures were obtained from these three areas with high ATP levels even after disinfection by protocol. Three keyboard and mouse sets located in the main station in NICU-A where all medical staff have access to and frequently use, were also included.

**Table 2:**
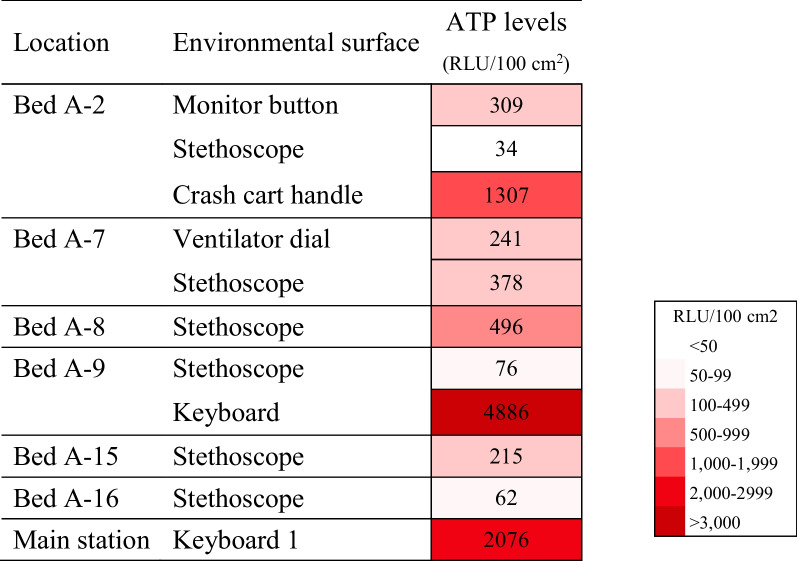
Random environmental surface ATP results during *E. faecium/S. capitis* outbreak in NICU-A after changing the disinfection method from Biospot® to Clinelle® and Safecide® (Intervention 2)

All surfaces with ≥ 100 RLU/ 100 cm^2^ after disinfection were considered contaminated and thus re-cleaned

ATP adenosine triphosphate; NICU neonatal intensive care unit; RLU, relative light units

#### 3) Investigation of transmission modes and identification of reservoirs

From the three high touch areas that maintained high levels of ATP after disinfection, *E. faecalis* was isolated from keyboard 1 of the main station, and *S. capitis* was isolated from keyboard of bed A-9 and crash cart handle by bed A-2.

**Fig. 3:**
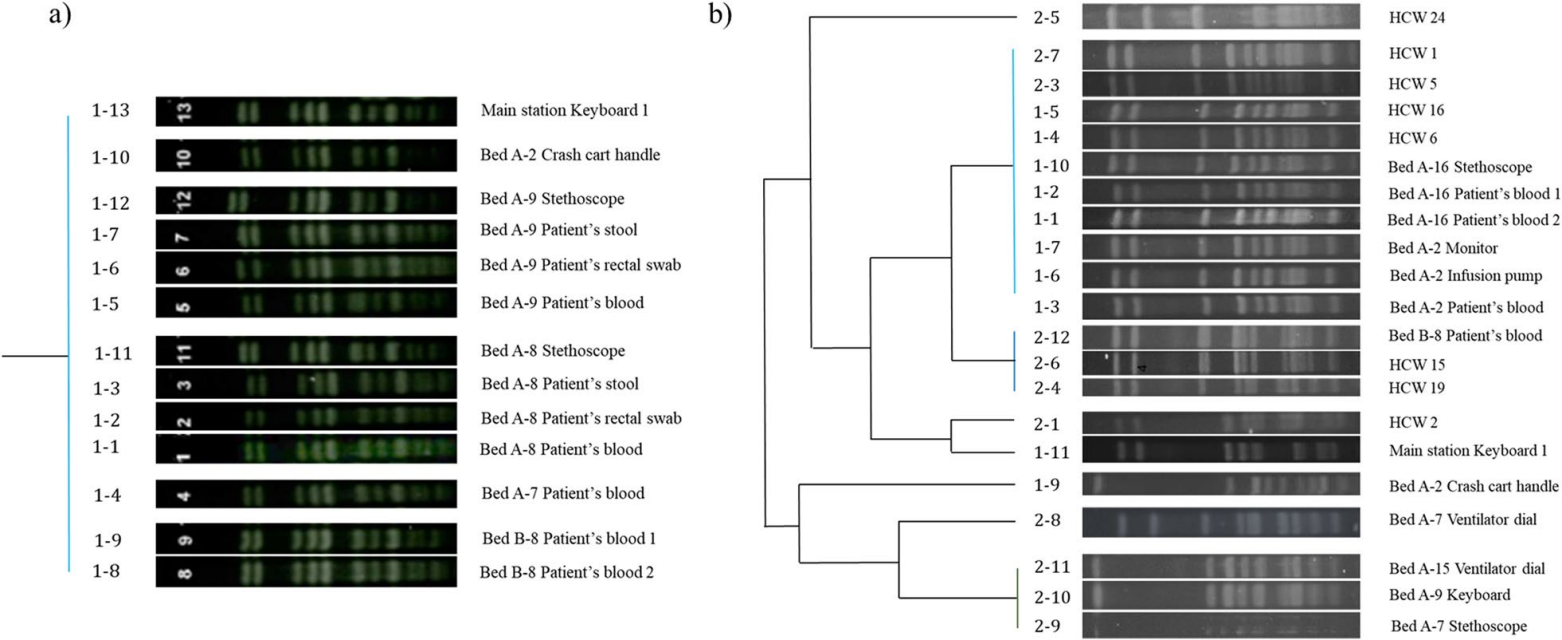
Dendrogram of **a** *E. faecium* PFGE types from all four patients (A-7, A-8, A-9, B-8), environmental cultures, and keyboard 1 of the main station showing identical strains, and **b** *S. capitis* PFGE types from all three patients (A-2, A-16, B-8), HCW’s hand cultures, and environmental cultures. Two different *S. capitis* strains were found to be the cause of sepsis in the patients. Results showed that 77.8% of the *S. capitis* identified from HCWs were the same strain that caused the *S. capitis* sepsis in patients A-2 and A-16. The remaining 22.2% were the same strain that caused sepsis in patient B-8. The environments surrounding patients A-2 and A-16 were also found to be contaminated with the same *S. capitis* strain. HCW, health care worker; PFGE, Pulsed-Field Gel Electrophoresis

Environment and HCW’s hand cultures showed that *E. faecium* was not isolated from hand cultures of HCWs, however, were isolated from environmental cultures (bed A-8, A-9 stethoscope, A-2 crash cart, and main station keyboard) and rectal cultures of the patients (2 out of 4: bed A-8, A-9). To find the genetic relatedness, all isolates underwent PFGE typing. *E. faecium* cultured from all four patients (A-7, A-8, A-9, B-8) and environmental cultures were found with a genotypically indistinguishable strain (Fig. 3a). Furthermore, *E. faecium* cultured from the main station’s keyboard 1 was also a genotypically indistinguishable strain (Fig. 3a, [1–13]). Because all medical staff had frequent usage of the main station keyboard 1, this was a possible reservoir for *E. faecium*.

Two different *S. capitis* strains were found to be the cause of sepsis in the patients. Genotypically indistinguishable strains were isolated from patients A-2 and A-16, whereas a different strain was identified from patient B-8 (Fig. 3b). Furthermore, *S. capitis* was isolated from 31.0% (n = 9/29) of the HCWs’ hands. Of these, 100% (n = 9/9) had similar antibiotic sensitivity profiles with *S. capitis* isolated from the patients with sepsis and therefore underwent PFGE typing (Fig. 3b). Results showed that 77.8% (n = 7/9) were found to be colonized with a genotypically indistinguishable strain causing *S. capitis* sepsis in patients A-2 and A-16. The remaining 22.2% (n = 2/9) were found with a genotypically indistinguishable strain that caused sepsis in patient B-8. The environments surrounding patients A-2 and A-16 were also found to be contaminated with the same *S. capitis* strain. The *S. capitis* isolated from keyboard of bed A-9 and crash cart handle by bed A-2A with high ATP levels after disinfection were a different strain. A common environmental reservoir was not found for *S. capitis* other than HCWs’ hands and environments immediately surrounding patients A-2 and A-16 (Fig. 3b).

With the possible reservoir of *E. faecium* identified as the main station’s keyboard 1, and mode of transmission for *S. capitis* as HCW’s hands, the 3rd intervention was incorporated. After the main station’s keyboard 1 and its mouse set were switched to a disinfectable keyboard and mouse, ATP levels were checked before and after disinfection to assess the effectiveness of the disinfection procedures. Compared to a conventional keyboard and mouse set located at the main station, keyboard 2 and mouse 2, the disinfectable keyboard and mouse set maintained low ATP levels prior to disinfection and were effectively decontaminated (Table 3). Based on these results, all keyboard and mouse sets in both NICU-A and B were switched to medical ones.

**Table 3:**
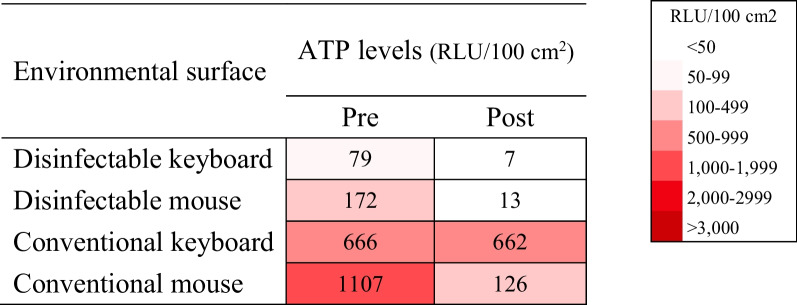
Comparison of random environmental surface ATP results immediately before and 1 h after disinfection in disinfectable keyboard and mouse versus conventional keyboard and mouse (Intervention 3)

All surfaces with ≥ 100 RLU/100 cm^2^ after disinfection were considered contaminated and thus re-cleaned

ATP adenosine triphosphate; NICU neonatal intensive care unit; RLU relative light units

### Assessing long-term effects of the infection control measures

**Fig. 4:**
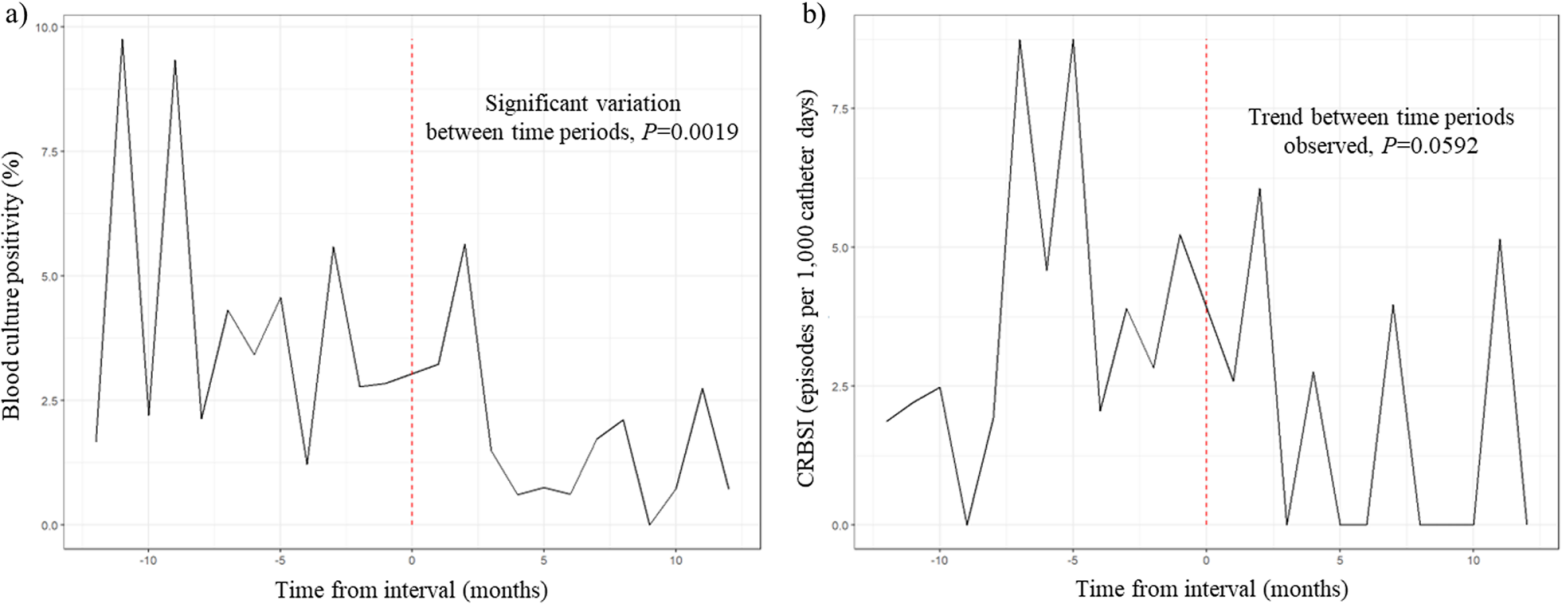
Interrupted time series analysis of monthly cases. **a** During the one-year period prior to the incorporation of the final infection control measures, blood culture positivity rate was 4.2% which fell to 1.6% during the 1st year after the final intervention (*P* = 0.0019). **b** The CRBSI rate was 3.7 episodes per 1000 catheter days during the one-year prior which fell to 2.0 episodes per 1000 catheter days during the 1st year after the final intervention (*P* = 0.0592). CRBSI, catheter-related blood stream infection

Prospective surveillance of hospital acquired sepsis and CRBSI rates were monitored up to 12 months after the third (final) intervention (Fig. 4). During the one-year period prior to the incorporation of the final infection control measures, blood culture positivity rate was 4.2% (n = 71/1698) which fell to 1.6% (n = 26/1590) during the 1^st^ year after the final intervention (*P* = 0.0019). Furthermore, the CRBSI rate was 3.7 episodes per 1000 catheter days during the one-year prior to the interventions, and fell to 2.0 episodes per 1000 catheter days during the 1^st^ year after the final intervention (*P* = 0.0592) (Fig. 4) (Monthly figures of blood culture positivity rate and CRBSI rate are on Additional file 1: B, C). During the one-year monitoring period after the final intervention, two cases of CRBSI caused by *S. capitis* occurred at 4 and 11 months after the final intervention.

## Discussion

Successful control of infectious disease outbreak in intensive care settings are important for minimizing its damaging effects. In this study, through ATP monitoring, high-touch areas where disinfection protocols failed to disinfect surfaces were identified and cultures were performed from these areas. By performing cultures on these surfaces, a non-medical computer device, keyboard 1, located at the main station of NICU-A was identified as one possible reservoir of *E. faecium*, which failed to decontaminate after intervention 1 and 2. Although areas of environmental surface contamination were identified for *S. capitis*, a common reservoir was not found. However, HCW’s hands were found to be colonized with a genotypically indistinguishable *S. capitis* strain that caused sepsis in the patients. Interventions including the disinfection of contaminated surfaces and reservoirs, as well as education and feedback on the importance of hand hygiene procedures based on the hand culture results of HCWs were effective in sustaining a decreased nosocomial infection rate in the NICU.

Successful outbreak management requires sterilization of not only the source or reservoirs but recognizing environmental factors, such as contaminated areas that may foster an outbreak (14, 15). However, finding these contaminated areas are difficult because bacterial contamination is not visible. Monitoring ATP bioluminescence levels have been shown to be a useful tool for identifying surfaces in hospitals and assessing the efficiency of cleaning procedures (4, 16, 17). In this study, through random ATP level testing, areas that were difficult to decontaminate or re-contaminate easily were discovered. In these areas, universal environmental disinfection procedures along with targeted disinfection of these easily re-contaminated areas were possible. Regardless of frequent disinfection and utilizing effective disinfectants, keyboard and mouse surfaces were found to be areas that were not only difficult to decontaminate, but also areas that were easily re-contaminated, serving as a source or reservoir of bacteria. Other studies have also shown that computer keyboards are contaminated with bacteria and can serve as a vehicle for transmission of pathogens to patients (18–20). These studies warrant that computers including keyboards should be disinfected frequently and hand hygiene procedures should be obeyed by the hospital staff before and after coming into contact with patients.

On the contrary, a systematic review of 75 studies involving 2804 computer devices reported that the overall proportion of contamination with skin commensals and potential pathogens was reported to range from 24 to 100%. However, this study concluded that the impact on transmission and nosocomial infection was unclear and warranted further studies to draw firm conclusions (21). Another study reported that whole genome sequencing showed patients with nosocomial infections had a different genotype than of those cultured from the keyboard. Therefore, although keyboard were a reservoir of multidrug-resistant organisms, they appeared unlikely to contribute to nosocomial infections (22). Our study contradicts this study, because through PFGE typing, we found that *E. faecium* isolated from patients with sepsis were genotypically the same strain as those isolated from a keyboard located in a distant area from patients. This shows that computer devices such as keyboards that are located in a distant area from patients can act as reservoirs, further highlighting the importance of environmental disinfection of high-touch areas by HCWs within all areas of the unit in preventing outbreaks by nosocomial pathogens. Furthermore, although we were unable to find *E. faecium* on the hands of employees, the hands must have also played a role in the transfer from keyboard to the patients.

Although many efforts were made to disinfect high-touch areas, the conventional keyboard and mouse sets were found to be difficult to decontaminate and were also easily re-contaminated. Therefore, all keyboards in the NICU were switched to disinfectable keyboards and mice, making disinfection procedures easier due to the absence of crevices and hidden surfaces between the keys. Furthermore, due to the smooth and regular surfaces of the disinfectable keyboards and mice, as well as surface material that allows lower rates of contamination, the long-term effects of the switch from conventional keyboards to disinfectable keyboards throughout the NICU contributed to lowering overall rates of CRBSIs up to one year after the final intervention.

The value of screening HCWs during an investigation of a nosocomial outbreak is yet unclear. A systematic review by Ulrich et al. reported that HCWs are rarely identified as the cause of outbreaks, and screening HCWs did not give any additional information (23, 24). However, in another study that reported an outbreak of *S. capitis* inside a NICU, *S. capitis* was cultured from HCW’s hand, and although this study did not perform genotyping, the study suggested that HCWs could be involved in inter-patient transmission of *S. capiti*s (25). Through genotyping, our study showed that HCWs carried genotypically indistinguishable *S. capitis* strains that caused sepsis in the patients, and therefore contributed to the transmission of *S capitis*.

Limitations of this study are that disinfectants used in this study and/or the timing of testing may have influenced ATP results. Secondly, ATP level monitoring was carried out on high-touch surfaces and not all hospital surfaces were included, providing possible bias in showing all contaminated areas. Also, not all HCW’s hand cultures were performed due to the nature of the random visits, which were deemed important to investigate colonization of the HCWs hands during duty. Finally, we were unable to include all sterile site infections or acquisition including carriage in this study, which limits representation of all transmission events.

## Conclusions

To conclude, this study showed that ATP monitoring was an effective tool in identifying areas that are difficult to disinfect. By culturing these areas, this study showed that non-medical devices such as keyboards can act as a reservoir of nosocomial pathogens. Furthermore, this study showed that colonization of potential pathogens in HCWs’ hands contributed to the transmission of nosocomial pathogens in NICUs. CRBSI rates before and after the interventions, which were disinfection of the reservoirs, adjustments to the disinfection policy and education on hand hygiene procedures, showed that the sustainability of the interventions was successful.

## Supplementary Information

**Additional file 1**. **Supplementary Material A**. Antibiotic susceptibility patterns of *E. faecium* and *S. capitis* cultured from the patients’ blood. **Supplementary Material B**. Monthly data on Blood culture positivity rate. **Supplementary Material C**. Monthly data on Episodes per 1,000 catheter days for all pathogens.

## Data Availability

The datasets used and/or analyzed during the current study are partially available from the corresponding author on reasonable request.

## Abbreviations

ATP: Adenosine triphosphate
NICU: Neonatal intensive care unit
PFGE: Pulsed-field gel electrophoresis
RLU: Relative light units
CRBSI: Catheter-related blood stream infection
HCW: Healthcare workers
HAI: Hospital acquired infections
IRB: Institutional Review Board
RDS: Respiratory distress syndrome
PDA: Patent ductus arteriosus
VRE: Vancomycin-resistant Enterococci
