# Supplementary material for: Using adenosine triphosphate bioluminescence level monitoring to identify bacterial reservoirs during two consecutive Enterococcus faecium and Staphylococcus capitis nosocomial infection outbreaks at a neonatal intensive care unit
Source: Antimicrob Resist Infect Control. 2023 Jul 13;12:68. doi: 10.1186/s13756-023-01273-5 (PMC10339505; doi:10.1186/s13756-023-01273-5)
Supplement: Supplementary file 1 — Additional file 1. Supplementary Material A. Antibiotic susceptibility patterns of E. faecium and S. capitis cultured from the patients’ blood. Supplementary Material B. Monthly data on Blood culture positivity rate. Supplementary Material C. Monthly data on Episodes per 1,000 catheter days for all pathogens. [file 13756_2023_1273_MOESM1_ESM.docx]

**Supplementary Material A**. Antibiotic susceptibility patterns of *E. faecium* and *S. capitis* cultured from the patients’ blood.

|  | Patients with *E. faecium* sepsis | | | |
| --- | --- | --- | --- | --- |
|  | A-8 | A-7 | A-8 | B-8 |
| Penicillin | S | S | S | S |
| Ampicillin | S | S | S | S |
| Ampicillin/sulbactam | S | S | S | S |
| Gentamicin High Level Resistance | S | S | S | S |
| Streptomicin High Level Resistance | S | S | S | S |
| Erythromycin | R | R | R | R |
| Quinupristin/dalfopristin | S | I | I | I |
| Linezolid | S | S | S | S |
| Teicoplanin | S | S | S | S |
| Vancomycin | S | S | S | S |
| Tigecycline | S | S | S | S |

|  | Patients with *S. capitis* sepsis | | |
| --- | --- | --- | --- |
|  | A-16 | A-2 | B-8 |
| Penicillin | R | R | R |
| Oxacillin | R | R | R |
| Gentamicin | I | I | R |
| Habekacin | S | S | S |
| Ciprofloxacin | R | R | I |
| Erythromycin | R | R | R |
| Telithromycin | S | S | R |
| Clindamycin | R | R | R |
| Linezolid | S | S | S |
| Teicoplanin | S | S | S |
| Vancomycin | S | S | S |
| Tetracycline | S | S | S |
| Tigecycline | S | S | S |
| Nitrofurantoin | S | S | S |
| Fusidic Acid | S | S | S |
| Rifampin | S | S | S |
| Trimethoprim/sulfamethoxazole | S | S | S |

**Supplementary Material B.** Monthly data on Blood culture positivity rate

| Months prior to final intervention | No. of positive blood cultures | Total no. of blood cultures performed | Positivity rate (%) |
| --- | --- | --- | --- |
| -12 | 2 | 121 | 1.7 |
| -11 | 12 | 123 | 9.8 |
| -10 | 2 | 91 | 2.2 |
| -9 | 14 | 150 | 9.3 |
| -8 | 3 | 141 | 2.1 |
| -7 | 5 | 116 | 4.3 |
| -6 | 4 | 117 | 3.4 |
| -5 | 8 | 175 | 4.6 |
| -4 | 2 | 164 | 1.2 |
| -3 | 10 | 179 | 5.6 |
| -2 | 5 | 180 | 2.8 |
| -1 | 4 | 141 | 2.8 |
| Total | 71 | 1698 | 4.2 |
|  |  |  |  |
| Months after final intervention | No. of positive blood cultures | Total no. of blood cultures performed | Positivity rate (%) |
| 1 | 4 | 124 | 3.2 |
| 2 | 7 | 124 | 5.6 |
| 3 | 2 | 134 | 1.5 |
| 4 | 1 | 166 | 0.6 |
| 5 | 1 | 134 | 0.7 |
| 6 | 1 | 161 | 0.6 |
| 7 | 2 | 116 | 1.7 |
| 8 | 2 | 95 | 2.1 |
| 9 | 0 | 113 | 0.0 |
| 10 | 1 | 137 | 0.7 |
| 11 | 4 | 146 | 2.7 |
| 12 | 1 | 140 | 0.7 |
| Total | 26 | 1590 | 1.6 |
| Blood culture positivity rate = No. of positive blood cultures/Total no. of Blood cultures performed * 100 | | | |

**Supplementary Material C.** Monthly data on Episodes per 1,000 catheter days for all pathogens

| Months prior to final intervention | CRBSI episodes | Catheter days | Episodes per 1,000 catheter days |
| --- | --- | --- | --- |
| -12 | 1 | 537 | 1.9 |
| -11 | 1 | 453 | 2.2 |
| -10 | 1 | 404 | 2.5 |
| -9 | 0 | 581 | 0.0 |
| -8 | 1 | 519 | 1.9 |
| -7 | 5 | 572 | 8.7 |
| -6 | 2 | 436 | 4.6 |
| -5 | 4 | 457 | 8.8 |
| -4 | 1 | 488 | 2.0 |
| -3 | 2 | 513 | 3.9 |
| -2 | 2 | 708 | 2.8 |
| -1 | 3 | 574 | 5.2 |
| Total | 23 | 6242 | 3.7 |
|  |  |  |  |
| Months after final intervention | CRBSI episodes | Catheter days | Episodes per 1,000 catheter days |
| 1 | 1 | 386 | 2.6 |
| 2 | 2 | 330 | 6.1 |
| 3 | 0 | 240 | 0.0 |
| 4 | 1 | 363 | 2.8 |
| 5 | 0 | 251 | 0.0 |
| 6 | 0 | 374 | 0.0 |
| 7 | 1 | 252 | 4.0 |
| 8 | 0 | 199 | 0.0 |
| 9 | 0 | 173 | 0.0 |
| 10 | 0 | 235 | 0.0 |
| 11 | 2 | 388 | 5.2 |
| 12 | 0 | 281 | 0.0 |
| Total | 7 | 3472 | 2.0 |

Episodes per 1,000 catheter days=CRBSI episodes/catheter days*1,000
